# SMARCA4-deficient undifferentiated carcinoma co-existing with primary endometrial gastric (or gastrointestinal) -type carcinoma: a case report

**DOI:** 10.3389/fonc.2025.1510930

**Published:** 2025-02-28

**Authors:** Fan Yang, Haixiao Fu, Xiang Zheng, Yuzhong Yang, Quyan Zhou, Yuanna Liang, Jinhua Zheng, Jing Lin

**Affiliations:** ^1^ Department of Pathology, Affiliated Hospital of Guilin Medical University, Guilin, China; ^2^ Department of Pathology, Guanyang County People’s Hospital, Guilin, China

**Keywords:** endometrial carcinoma, UEC, DEC, PEGT-carcinoma, SMARCA4-deficient

## Abstract

Dedifferentiated endometrial carcinoma (DEC) is a rare and highly malignant endometrial tumor consisting of both undifferentiated endometrial carcinoma (UEC) and differentiated components (that are typically grade 1 or 2 endometrioid carcinomas). By contrast, the coexistence of UEC with a high-grade endometrial carcinoma has not been well reported. Primary endometrial gastric (gastrointestinal)-type carcinoma (PEGT-carcinoma) is a newly classified and rare type of female genital tract cancer, this neoplasm belongs to high-grade endometrial carcinoma and tends to have a poor outcome. In this study, we reported an atypical case of DEC composed of SMARCA4-deficient UEC and PEGT-carcinoma and reviewed its clinicopathological features.

## Introduction

The fifth edition of the World Health Organization (WHO) classification includes a novel and rare endometrial cancer subtype: primary gastric-type carcinoma (PEGT-carcinoma), characterized by gastric/mucinous gastrointestinal features. Although morphologically less aggressive, PEGT-carcinoma is a high-grade malignancy with poor prognosis (1–3). Dedifferentiated endometrial carcinoma (DEC) is a rare, highly malignant variant, featuring undifferentiated endometrial carcinoma (UEC) alongside low-grade endometrioid carcinoma (4–6). UEC is identified by monotypic tumor cells lacking glandular or squamous differentiation, while the differentiated component is usually International Federation of Gynecology and Obstetrics (FIGO) grade 1 or 2 (4–8). We present an unusual DEC case with SMARCA4-deficient UEC and PEGT-carcinoma, reviewing its clinicopathological profile.

## Case presentation

**Figure 1:**
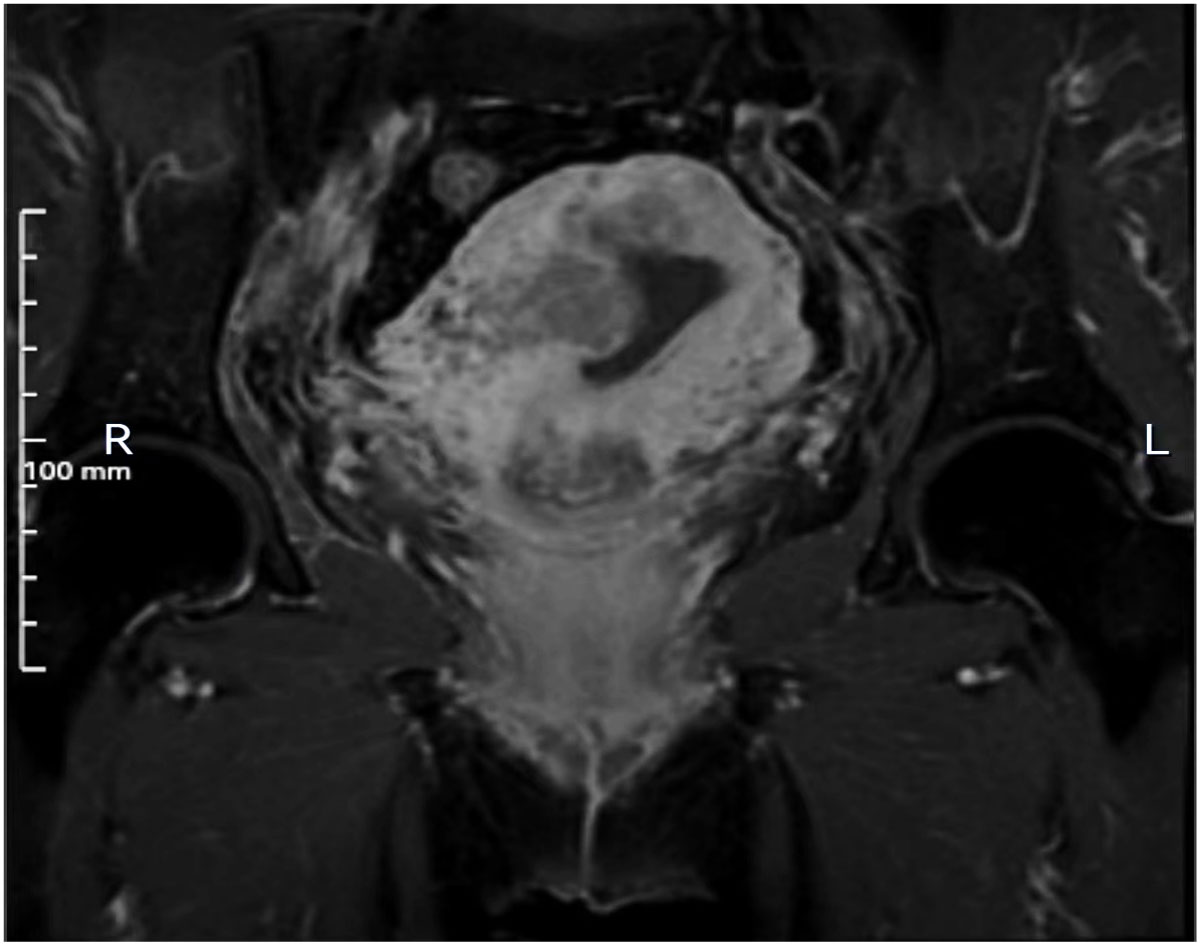
MRI imaging. MRI shows the lesion is situated on the right lateral wall of the uterus and exhibits inhomogeneous enhancement.

A 64-year-old postmenopausal woman presented with irregular vaginal bleeding for 20 days. She had no cancer family history. Physical examination revealed vaginal blood accumulation with no cervical or uterine abnormalities. Ultrasonography indicated uterine fibroids and intracavitary blood, suggesting a possible tumor. Her Serum tumor markers of CEA, AFP, CA-125, CA-153 and CA-199 were within normal limits, and sex hormone levels were consistent with menopause. MRI showed a uterine lesion with heterogeneous enhancement and lymph node enlargement in the pelvic region (**Figure 1**). Gastroenteroscopy found no masses. Pathological analysis post-curettage confirmed mucinous adenocarcinoma, and subsequent radical surgery was performed.

**Figure 2:**
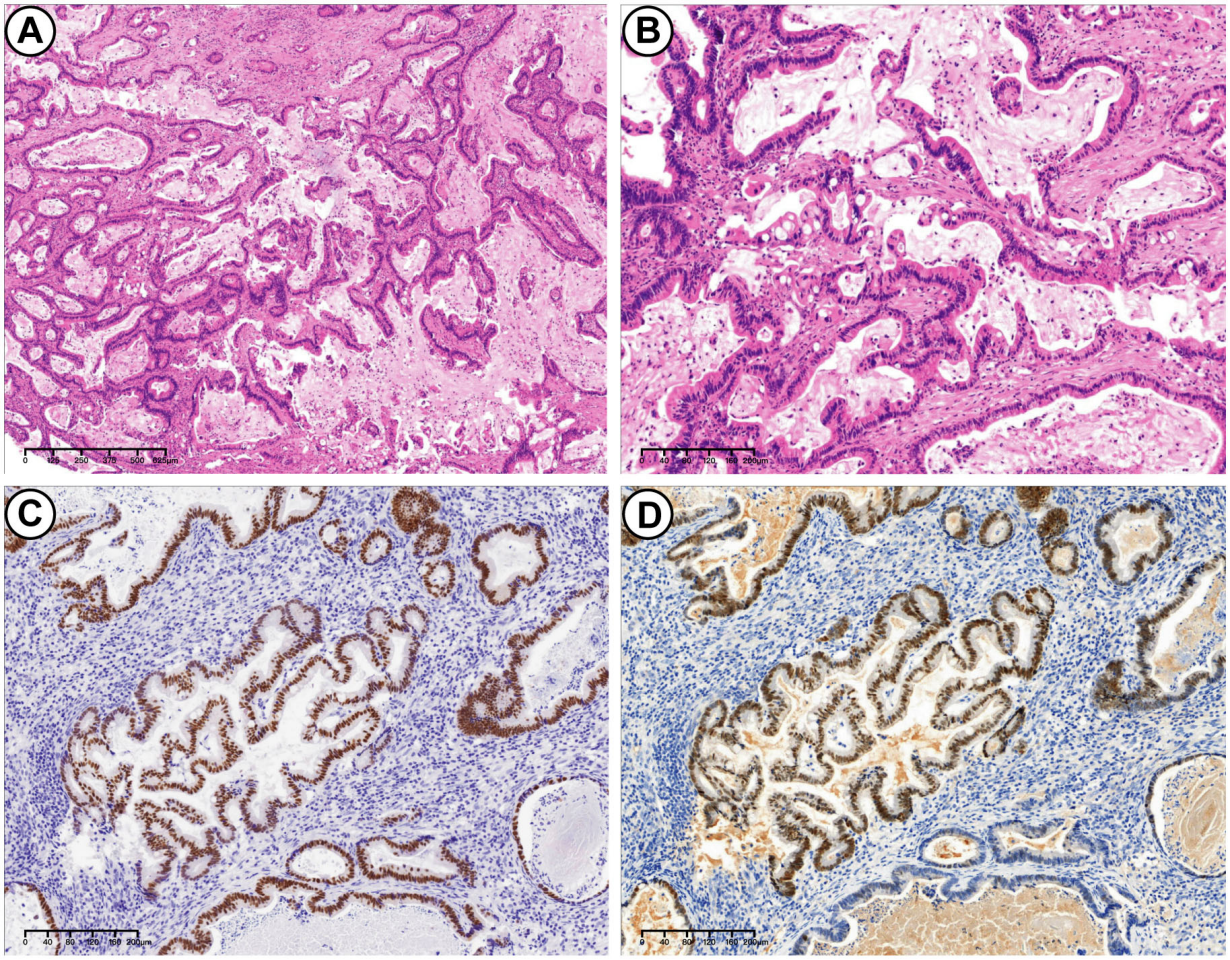
PEGT-carcinoma. **(A)** H&E stains. Irregular glands surrounded by a significant amount of mucus. Some glands exhibited dilated, sieve-like structures, while others displayed papillary structures (4×). **(B)** H&E stains. These glands were lined with a single or complex layer of columnar epithelium, with cup-shaped cells visible between the columnar epitheliums. The tumor cells were irregularly arranged (10×). **(C)** Immunohistochemical stains: positive for CDX2 (10×). **(D)** Immunohistochemical stains: partially positive for SATB2 (10×).

Microscopically, the tumor exhibits a dual-component structure. The first component is PEGT-carcinoma, characterized by irregular, mucus-filled glands, some of which display dilated sieve-like or papillary formations. The columnar epithelium is either single or multi-layered, featuring cup-shaped cells. Tumor cells are distinguished by large nuclei, prominent nucleoli, and high mitotic activity (**Figure 2**). Immunohistochemical analysis revealed positivity for CDX2, SATB2, CK7, CK20, PMS2, MLH1, MSH2, MSH6, and wild-type p53, while showing negativity for p16, Pax-8, ER, PR, Muc-6, Muc-5AC and claudin 18.2. The Ki67 proliferation index was approximately 80%. Alcian Blue-Periodic Acid-Schiff (AB-PAS) staining confirmed the presence of mucus and goblet cells.

**Figure 3:**
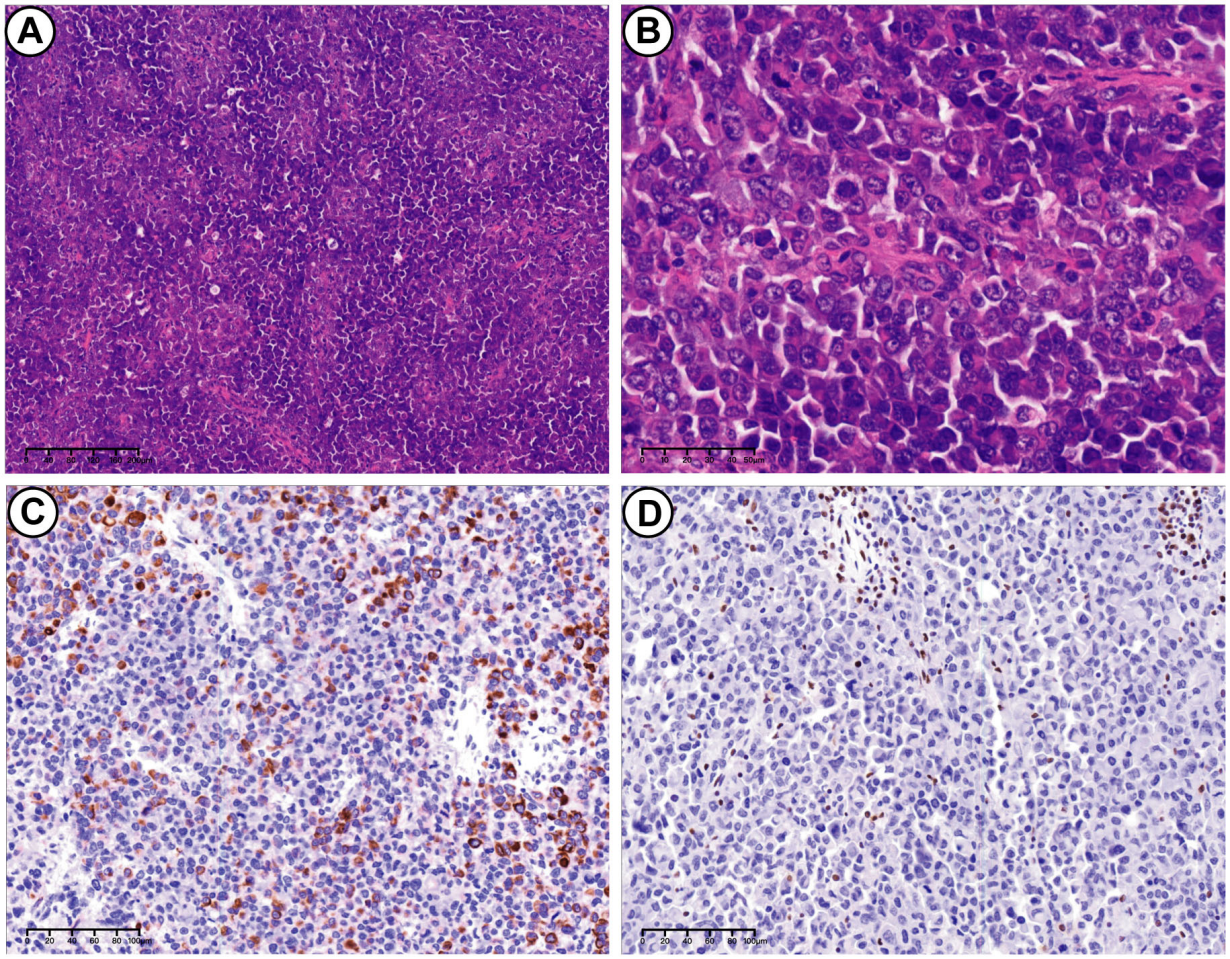
UEC. **(A)** H&E stains. UEC consisted of neoplastic cells arranged in diffuse sheets (10×). **(B)** H&E stains. Tumor characterized by enlarged and markedly atypical nuclei, and moderate to abundant cytoplasm (40×). **(C)** Immunohistochemical stains. Partially positive for CK (20×). **(D)** Immunohistochemical stains. Loss of SMARCA4(BRG1) expression in UEC (20×).

Secondly, the UEC exhibited a lack of glandular differentiation, with neoplastic cells arranged in diffuse sheets. These cells displayed enlarged, atypical nuclei, prominent nucleoli, and moderate to abundant cytoplasm. Rhabdoid morphology was observed in certain regions. A high mitotic rate and extensive necrosis were present, along with evidence of vascular invasion (**Figure 3**). Immunohistochemical analysis demonstrated positivity for vimentin, INI-1, PMS2, MLH1, MSH2, MSH6, and wild-type p53. CK showed partial and weak positivity, whereas BRG1, Mammaglobin, S100, Pax-8, p16, and ER were negative. The Ki-67 proliferation index was approximately 60%. The tumor components were generally distinct, with minimal intermingling. The specimen was estimated to contain 20% PEGT-carcinoma and 80% UEC.

Concurrently, a panel developed by Geneseeq (Nanjing, China) targeting 506 cancer-associated genes was utilized to conduct next-generation sequencing (NGS) analysis on the bipartite tumor tissue. In accordance with the manufacturer’s guidelines, the post-capture library was assessed using the Agilent Technologies 2100 Bioanalyzer (Agilent, Santa Clara, CA, USA). Somatic mutations were identified utilizing VarScan2. Annotation was performed with ANNOVAR, referencing the Human Genome version 19 (hg19) and incorporating the 2014 versions of standard databases and functional prediction tools. The NGS analysis results indicated that 8 variants were unique to the PEGT-carcinoma component, 7 variants were specific to the UEC component, and 7 variants were common to both components (**Table 1**). Notably, a nonsense mutation (p.Y769*) in the SMARCA4 gene was identified within the UEC component, representing a particularly significant finding. Both tumor components exhibited a substantial mutation burden, with the PEGT-carcinoma component presenting 16.5 mutations per megabase (Muts/Mb) and the UEC component displaying 11.6 Muts/Mb. Notably, no POLE mutations were identified in either tumor component. Furthermore, both components demonstrated microsatellite stability. And no germline mutations were detected.

**Table 1:** Next-generation sequencing of two components of DEC.

Group	PEGT-carcinoma				UEC			
	Gene	Mutation	Mutation classification	Mutation frequency	Gene	Mutation	Mutation classification	Mutation frequency
Unique	AKT1	c.49G>A(p.E17K)	Missense	3.54%	ATRX	c.1588G>C(p.E530Q)	Missense	44.84%
	BAP1	c.1730-1G>A	Splicing	3.33%	BCR	c.3223G>A(p.V1075M)	Missense	7.86%
	FAT1	c.10207-1G>T	Splicing	7.69%	DOT1L	c.454A>T(p.K152*)	Nonsense	48.54%
	KEAP1	c.1525G>A(p.G509R)	Missense	8.25%	EP300	c.4373C>A(p.P1458H)	Missense	60.53%
	MDM4	c.1100C>T(p.S367L)	Missense	20.67%	IKBKE	c.250A>G(p.K84E)	Missense	18.71%
	NF2	c.1300G>T(p.E434*)	Nonsense	1.77%	MCL1	/	AP	CN:20.76
	PBRM1	c.2965+1G>A	Splicing	8.54%	MYC	/	AP	CN:8.26
		c.996-1G>A	Splicing	3.85%				
		c.1175 1176del(p.T392sfs*2)	Splicing	1.08%				
	PRKN	c.147G>T(P.E49D)	Missense	2.23%				
Common	BCL2L11	c.395+1479_394+4381del	LOH	/	BCL2L11	c.395+1479_394+4381del	LOH	/
	CHD4	c.3369G>T(p.L1123F)	Missense	24.37%	CHD4	c.3369G>T(p.L1123F)	Missense	20.36%
	DPYD	c.1627A>G(p.I543V)	SNP	/	DPYD	c.1627A>G(p.I543V)	SNP	/
	GSTM1	/	HD	/	GSTM1	/	HD	/
	SMARCA4	c.3476G>A(p.G1159E)	Missense	9.20%	SMARCA4	c.2307C>G(p.Y769*)	Nonsense	60.13%
		c.2573C>T(p.T858M)	Missense	1.90%				
	STK11	c.82 248delinsG(p.R28Gfs*13)	FS	8.31%	STK11	c.298C>T(p.Q100*)	Nonsense	50.20%
		c.810del(p.s271Afs*16)	Missense	7.28%				
		c.526G>A(p.D176N)	FS	3.74%				
		c.298C>T(p.Q100*)	Nonsense	0.48%				
	TP53	c.524G>A(p.R175H)	Missense	0.94%	TP53	c.524G>A(p.R175H)	Missense	53.94%

/, null; LOH, Loss of Heterozygosity; SNP, Single Nucleotide Polymorphism; HD, Homozygous Deletion; FS, Frame Shift Mutation; AP, Amplification.

The patient declined chemotherapy post-surgery, opting for traditional Chinese medicine as a self-treatment method. However, a follow-up pelvic MRI on December 15, 2023, indicated multiple enlarged pelvic lymph nodes, suggesting cancer metastasis. Despite this, the patient was still alive at the time of report preparation.

## Discussion

In an extensive review of 25 cases exhibiting the simultaneous presence of low-grade endometrioid carcinoma and UEC, Silva et al. introduced the term “DEC” to describe this specific tumor entity (5). The 2020 WHO classification defines DEC as a neoplasm comprising UEC alongside FIGO grade 1-2 endometrioid carcinoma. UEC, which constitutes approximately 2% of endometrial cancers (9), is histologically characterized by its discohesive cells organized in sheets without a nested or glandular pattern, and its rarity of keratinization (4).

Recurrent genetic mutations have been identified in both DEC and UEC. These mutations often involve inactivating alterations in core SWI/SNF complex proteins. Mutations in SMARCA4 (BRG1), SMARCB1 (INI1), ARID1A, and ARID1B have been implicated in approximately two-thirds of DEC cases and half of UEC cases (10–13). The mutation frequencies for SMARCA4 and SMARCB1 were observed to range from 13% to 100% and 4% to 15%, respectively (14). In our case, immunohistochemistry staining revealed a deficiency of BRG1 in the UEC component, and this finding was further supported by the confirmation of a nonsense mutation in SMARCA4 (p.Y769*). Additionally, TP53 mutations present in approximately 26% to 38% of UEC/DEC cases (9), In our case, TP53 were identified as missense mutation in UEC parts through NGS analysis, despite immunohistochemistry suggesting a wild-type p53.

PEGT-carcinoma is infrequently reported, with its morphological characteristics defined by glands composed of mucin-secreting epithelium, which may include goblet cells (2). These glands typically exhibit nuclei with low-grade atypia. It is essential to exclude the possibility of a cervical origin or metastasis from the gastrointestinal tract when diagnosing PEGT-carcinoma (1). Additionally, PEGT-carcinoma is marked by the absence of an endometrioid component (2, 15). In this study, the integration of imaging data from MRI and gastrointestinal endoscopy, combined with the absence of both personal and familial medical history of gastrointestinal tumors in the patient, facilitated comprehensive examinations that excluded the presence of additional tumors, thereby supporting the hypothesis of a primary endometrial origin. The tumor demonstrated features consistent with an intestinal-type adenocarcinoma, without any evidence of gastric-type adenocarcinoma. Nevertheless, following the nomenclature established by the WHO classification, this case is categorized as PEGT-carcinoma.

DEC often presents with a distinct spatial arrangement, where the differentiated component is superficial and adjacent to the endometrial cavity, while the undifferentiated component is deeper within the endometrium and myometrium (4, 5). This pattern was observed in our case as well.

It is noteworthy that, despite presenting with low-grade morphological characteristics, PEGT-carcinoma frequently demonstrates aggressive behavior. This is evidenced by features such as lymphovascular invasion, deep myometrial invasion, and a tendency for recurrence even when identified at a low FIGO stage (1, 3, 15, 16). Although DEC is generally characterized by the coexistence of UEC and low-grade endometrioid carcinoma, there have been documented instances of UEC arising within the context of high-grade endometrial carcinoma, referred to as DEC-HG (7, 17). Ekaterina Olkhov-Mitsel et al. conducted genomic sequencing and immunohistochemical analyses on seven cases of DEC-HG and four cases of DEC associated with low-grade endometrial carcinoma (DEC-LG). Their findings revealed comparable mutation rates and types across both DEC-HG and DEC-LG, leading them to propose an expansion of the definition of DEC to include DEC-HG (18). In support of this proposition, the tumor in our case exhibited a mutation pattern akin to that typically observed in conventional DEC.

In conclusion, DEC, particularly when its differentiated component is PEGT-carcinoma, is a rare variant of endometrial carcinoma. Considering the presence of high-grade differentiated carcinoma in DEC, it is recommended to broaden the diagnostic criteria for DEC. This inclusive approach aids in more precise diagnosis and, consequently, in optimizing treatment strategies for patients.

## Data Availability

The original contributions presented in the study are included in the article/supplementary material. Further inquiries can be directed to the corresponding authors.

## References

[1] Bragantini E Angelico G Disanto MG Magri E Maccio L Barbareschi M. Gastric (gastrointestinal)-type endometrial adenocarcinoma presenting as a solitary endometrial polyp: a case report and literature review on a novel and potentially aggressive endometrial cancer histotype. Pathologica. (2023) 115:227–31. doi: 10.32074/1591-951X-870 PMC10688243 37711039

[2] Dridi M Peoc’h M Karpathiou G. Primary endometrial gastric (gastro-intestinal)-type carcinoma: A practical approach. Pathol Res Pract. (2023) 241:154271. doi: 10.1016/j.prp.2022.154271 36502736

[3] Kawakami F Yamaguchi K Minamiguchi S Sudo T Hirose T Teramoto N. Endometrial gastric-type mucinous carcinoma: A clinicopathological study of an unfavorable histological type of endometrial carcinoma. Pathol Int. (2023) 73:609–11. doi: 10.1111/pin.13376 37671817

[4] Altrabulsi B Malpica A Deavers MT Bodurka DC Broaddus R Silva EG. Undifferentiated carcinoma of the endometrium. Am J Surg Pathol. (2005) 29:1316–21. doi: 10.1097/01.pas.0000171003.72352.9a 16160474

[5] Silva EG Deavers MT Bodurka DC Malpica A. Association of low-grade endometrioid carcinoma of the uterus and ovary with undifferentiated carcinoma: a new type of dedifferentiated carcinoma? Int J Gynecol Pathol. (2006) 25:52–8. doi: 10.1097/01.pgp.0000183048.22588.18 16306785

[6] Kuhn E Ayhan A Bahadirli-Talbott A Zhao C Shih Ie M. Molecular characterization of undifferentiated carcinoma associated with endometrioid carcinoma. Am J Surg Pathol. (2014) 38:660–5. doi: 10.1097/PAS.0000000000000166 24451280

[7] Busca A Parra-Herran C Nofech-Mozes S Djordjevic B Ismiil N Cesari M. Undifferentiated endometrial carcinoma arising in the background of high-grade endometrial carcinoma - Expanding the definition of dedifferentiated endometrial carcinoma. Histopathology. (2020) 77:769–80. doi: 10.1111/his.14186 32557836

[8] Berek JS Matias-Guiu X Creutzberg C Fotopoulou C Gaffney D Kehoe S. FIGO staging of endometrial cancer: 2023. J Gynecol Oncol. (2023) 34:e85. doi: 10.3802/jgo.2023.34.e85 37593813 PMC10482588

[9] Tafe LJ Garg K Chew I Tornos C Soslow RA. Endometrial and ovarian carcinomas with undifferentiated components: clinically aggressive and frequently underrecognized neoplasms. Mod Pathol. (2010) 23:781–9. doi: 10.1038/modpathol.2010.41 20305618

[10] Strehl JD Wachter DL Fiedler J Heimerl E Beckmann MW Hartmann A. Pattern of SMARCB1 (INI1) and SMARCA4 (BRG1) in poorly differentiated endometrioid adenocarcinoma of the uterus: analysis of a series with emphasis on a novel SMARCA4-deficient dedifferentiated rhabdoid variant. Ann Diagn Pathol. (2015) 19:198–202. doi: 10.1016/j.anndiagpath.2015.04.001 25920939

[11] Karnezis AN Hoang LN Coatham M Ravn S Almadani N Tessier-Cloutier B. Loss of switch/sucrose non-fermenting complex protein expression is associated with dedifferentiation in endometrial carcinomas. Mod Pathol. (2016) 29:302–14. doi: 10.1038/modpathol.2015.155 PMC4980656 26743474

[12] Ramalingam P Croce S McCluggage WG. Loss of expression of SMARCA4 (BRG1), SMARCA2 (BRM) and SMARCB1 (INI1) in undifferentiated carcinoma of the endometrium is not uncommon and is not always associated with rhabdoid morphology. Histopathology. (2017) 70:359–66. doi: 10.1111/his.13091 27656868

[13] Coatham M Li X Karnezis AN Hoang LN Tessier-Cloutier B Meng B. Concurrent ARID1A and ARID1B inactivation in endometrial and ovarian dedifferentiated carcinomas. Mod Pathol. (2016) 29:1586–93. doi: 10.1038/modpathol.2016.156 27562491

[14] Kaur R Mehta J Borges AM. Role of SMARCA4 (BRG1) and SMARCB1 (INI1) in dedifferentiated endometrial carcinoma with paradoxical aberrant expression of MMR in the well-differentiated component: A case report and review of the literature. Int J Surg Pathol. (2021) 29:571–7. doi: 10.1177/1066896920959453 32940101

[15] Wong RW Ralte A Grondin K Talia KL McCluggage WG. Endometrial gastric (Gastrointestinal)-type mucinous lesions: report of a series illustrating the spectrum of benign and Malignant lesions. Am J Surg Pathol. (2020) 44:406–19. doi: 10.1097/PAS.0000000000001381 31567280

[16] Fujiwara M Longacre TA. Low-grade mucinous adenocarcinoma of the uterine corpus: a rare and deceptively bland form of endometrial carcinoma. Am J Surg Pathol. (2011) 35:537–44. doi: 10.1097/PAS.0b013e31820f1cc2 21378544

[17] Lee SE Park HY Shim SH Kim WY. Dedifferentiated carcinoma with clear cell carcinoma of the endometrium: A case report. Pathol Int. (2017) 67:472–6. doi: 10.1111/pin.12557 28667682

[18] Olkhov-Mitsel E Busca A Parra-Herran C Amemiya Y Nofech-Mozes S Djordjevic B. Genomic profiling of dedifferentiated endometrial carcinomas arising in the background of high-grade carcinoma: a targeted next-generation sequencing study. Histopathology. (2023) 83:366–75. doi: 10.1111/his.14938 37222195

